# Nutritional Modulation of the Gut Microbiome in Relation to Prenatal Lead-Induced Neurotoxicity: A Review

**DOI:** 10.3390/nu17233700

**Published:** 2025-11-26

**Authors:** Shoshannah Eggers, Kiran P. Nagdeo, Kshitij Sachdev, Delaney Robinson, Andrea L. Deierlein, Jamil M. Lane, Chris Gennings, Ryan W. Walker, Linda Snetselaar, Nichole Nidey, Elizabeth E. O’Neal, Vishal Midya

**Affiliations:** 1 Department of Epidemiology, University of Iowa College of Public Health, 145 N. Riverside Drive, Iowa City, IA 52242, USA; shoshannah-eggers@uiowa.edu (S.E.);; 2 Department of Epidemiology, New York University School of Global Public Health, 708 Broadway, New York, NY 10003, USA; 3 Department of Surgery, University of Iowa Carver College of Medicine, Iowa City, IA 52242, USA; 4 Department of Public Health Nutrition, New York University School of Global Public Health, 708 Broadway, New York, NY 10003, USA; 5 Department of Environmental Medicine, Icahn School of Medicine at Mount Sinai, 1 Gustave L. Levy Place, New York, NY 10029, USA; 6 Department of Community and Behavioral Health, University of Iowa College of Public Health, 145 N. Riverside Drive, Iowa City, IA 52242, USA

**Keywords:** gut-brain axis, Developmental Origins of Health and Disease (DOHaD), diet, narrative review

## Abstract

Prenatal exposure to lead (Pb) is a well-established risk factor for adverse neurodevelopmental outcomes. Despite its recognized risks, prenatal Pb exposure remains largely unregulated and poorly addressed in public health policy. Evidence suggests that the gut microbiome may mediate the neurotoxic effects of metals, offering a potential target for intervention. Here we discuss how nutritional factors, particularly those influencing the gut microbiome, may reduce the neurotoxicity of prenatal Pb exposure.

## 1. Introduction

Lead (Pb) is one of the most studied environmental toxins, yet Pb exposure remains a pervasive public health concern. Pb is a potent neurotoxin that can cross the placental barrier, exposing the fetus to its harmful effects even before birth [1]. Even low-level maternal exposure can result in fetal accumulation [2]. Extensive research has demonstrated Pb to be a teratogen that can lead to significant neurological issues, including cognitive deficits, attention disorders, and behavioral disorders [3]. Children exposed to Pb are at an increased risk of developing lower IQ, learning disabilities, and other cognitive impairments that can persist throughout life [4]. Pb exposure has also been linked to an elevated risk of neuropsychiatric conditions such as attention-deficit/hyperactivity disorder (ADHD), autism spectrum disorder (ASD), and mood disorders like anxiety and depression [5]. These effects are often irreversible, making early-life exposure especially concerning.

Even though leaded gasoline was banned in the US decades ago, exposure to Pb remains widespread and comes from several sources, including industry emissions, old housing and water line infrastructure, Pb solder in electronics, polluted soil and water, and older products like batteries and lead-based paint [6]. While Pb exposure in children is closely monitored during well child visits, Pb exposure in women during pregnancy remains largely unmonitored. While both the Center for Disease Control and Prevention (CDC) and the Occupational Safety and Health Administration (OSHA) provide guidance on Pb exposure during pregnancy, particularly for pregnant workers, these recommendations are advisory and lack enforceable screening mandates or legally binding protections [7]. There is a pressing need for comprehensive prenatal Pb monitoring and regulatory guidelines to safeguard maternal and fetal health.

**Figure 1:**
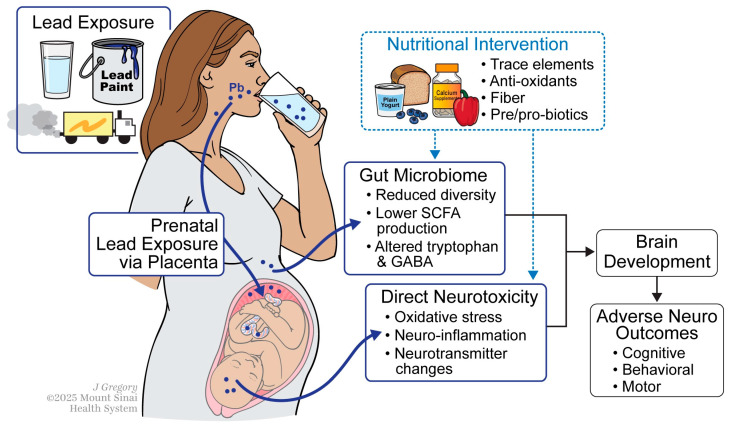
General Overview of Mechanisms. Illustration of mechanisms connecting prenatal lead (Pb) exposure to adverse neurological outcomes in children. Maternal exposure to Pb from environmental pollution can transfer to a fetus through the placenta, leading to neurotoxicity through oxidative stress, neuro-inflammation, and altered neurotransmitter levels. Targeted dietary interventions may reduce Pb induced neurotoxicity to the fetus by reducing Pb absorption and toxicity directly and through the gut microbiome.

The lack of monitoring and regulatory oversight for Pb exposure in pregnant women highlights the urgent need for interventions that can mitigate the risks associated with prenatal exposure. This narrative review aims to provide an overview of the gut microbiome as a key component of nutritional interventions against adverse neurodevelopment upon early-life Pb exposure (Figure 1). We first summarize mechanisms by which lead disrupts neurodevelopmental processes, followed by evidence positioning the gut microbiome as a mediator of lead neurotoxicity. We then discuss nutritional interventions that may modulate the microbiome to reduce lead toxicity and conclude by highlighting gaps in the literature and proposing future directions. By synthesizing findings from preclinical and clinical studies, we explore the complex interplay among these factors and examine potential therapeutic interventions for mitigating adverse neurodevelopmental outcomes.

## 2. Literature Identification

This narrative review drew articles primarily from PubMed and Google Scholar. The search included terms related to lead exposure, neurodevelopment, nutrition, and the gut microbiome, including “neurodevelopment,” “gut microbiome,” “maternal diet,” “pregnancy,” “prenatal,” “lead exposure,” “nutrition,” and “intervention” in combination with Boolean operators to refine results. Additional records were identified through citations of included studies.

## 3. Mechanisms of Lead Exposure and Prenatal Neurodevelopment

The mechanisms by which Pb exerts its toxic effects are diverse and complex. One of the primary mechanisms is Pb’s ability to mimic and substitute for essential metals such as calcium (Ca), zinc (Zn), and iron (Fe) within the body. For example, Pb can replace Ca in biological systems, disrupting processes like neurotransmitter release, and accumulating in bones across the lifespan, and can be re-introduced into the blood stream during bone remodeling. In the nervous system, Pb can cross the blood–brain barrier and interfere with synaptic transmission, leading to impaired cognitive function and neurodevelopmental issues [5]. Pb can be particularly neurotoxic during fetal development [8], and previous maternal exposures may contribute to even higher fetal exposure due to bone remodeling during pregnancy that releases stored Pb back into plasma [9]. Pb induces oxidative stress by generating reactive oxygen species and depleting antioxidant defenses, which can result in cellular damage and apoptosis across various tissues, including the brain [10]. Pb also disrupts heme synthesis, leading to anemia and impairing the body’s ability to effectively transport oxygen throughout the body, including the nervous system. Prenatal exposures may have even larger impacts on brain development as the trajectory of development, including key stages such as neurogenesis, synaptogenesis, and myelination, is initiated in utero, with potentially cascading impacts. Early-life stressors, including environmental, nutritional, and microbial exposures, can alter the development of the amygdala and prefrontal cortex, particularly during critical periods of development, impacting emotional regulation and decision-making [11].

Prenatal Pb exposure has been robustly and consistently linked to adverse neurodevelopmental outcomes in children. Birth cohorts across multiple countries have identified elevated maternal blood Pb levels during pregnancy to be associated with delays in cognitive development and lower intelligence [12,13]. Even low-level prenatal Pb exposure has been associated with reduced cognitive performance and IQ scores in early childhood, with no identifiable threshold for safe exposure [14]. Further investigation has shown associations with prenatal exposures and neurological health beyond cognition, including deficits in attention, memory, executive function, and behavioral problems in children [15]. Additional studies have shown more nuanced relationships, including non-linear and mixed metal effects [16]. This strong body of evidence emphasizes the heightened vulnerability of the developing brain to Pb and highlights the need for early interventions aimed at minimizing maternal Pb exposure during pregnancy.

## 4. The Gut Microbiome as a Mediator

The human gut houses a diverse array of microbes, collectively referred to as the gut microbiome, which plays a pivotal role in human health, impacting metabolism, nutrient absorption, gut motility, immune responses, and brain development [17]. Dysbiosis, or imbalance in the gut microbiome, has been linked to gastrointestinal ailments, as well as broader systemic conditions, including neurodevelopmental and neuropsychiatric conditions [18,19]. Pb exposure significantly impacts the composition and function of the gut microbiome, which may mediate Pb’s broader toxic effects on health [20,21], evidence of which is summarized in Table 1.

In animal models, Pb exposure reduced microbial diversity and particularly beneficial bacteria like *Lactobacillus*, while it increased potentially harmful genera such as *Clostridium* and *Oscillibacter*, which are associated with inflammation and metabolic disorders [22]. Similarly, early-life Pb exposure caused long-lasting alterations in the gut microbiome, which were linked to changes in metabolic profiles in adulthood [23]. In human studies, our group found that urinary Pb concentrations in adults were associated with increased α-diversity and β-diversity of the gut microbiome, and a higher likelihood of Proteobacteria colonization, a common marker of gut dysbiosis [24], even at relatively low levels of exposure [25]. Others have found that blood Pb level is correlated with Gammaproteobacteria and a few other genera in children with high exposure to several heavy metals [26]. Another study showed that significant alterations in 114 metabolic pathways within the gut microbiome were associated with stool Pb level in children [27]. Prenatal Pb exposure has been increasingly recognized for its potential to disrupt the developing gut microbiome as well. A study of prenatal Pb exposure measured through baby teeth found that higher prenatal Pb levels were associated with altered gut fungal communities and several bacterial taxa in infants at one and six months of age [28]. Another study from our group demonstrated a negative association between maternal blood Pb levels during pregnancy and child gut microbiome at 9–11 years of age, including reduced diversity and altered abundance of specific taxa like *Ruminococcus gnavus* and *Bifidobacterium longum*, both of which tend to benefit gut health [29]. These findings underscore the potentially lasting effects of Pb on gut microbiome composition and function.

The gut–brain axis, a bidirectional communication network between the gut and the brain, is heavily influenced by the gut microbiome [30]. This link is evidenced by studies showing that alterations in gut bacteria and their functions can affect neuropsychiatric outcomes, including conditions such as depression, anxiety, and ASD [19], as well as neurological diseases, like multiple sclerosis, Alzheimer’s Disease, and Parkinson’s Disease [18]. As with environmental exposures, the prenatal period can be a critical time for microbial exposures to impact the dynamic process of brain development. Although there is no consensus on the existence of the gut microbiome in utero, prenatal exposures can influence the development of the gut microbiome and its impact on the brain through shifts in the maternal microbiome and programming of immune development in utero [31]. Previous studies have investigated links between prenatal microbial exposures, like antibiotics, and found negative associations with neurological outcomes in children [32]. Mouse models have shown that the maternal microbiome during pregnancy promotes fetal brain development through microbial metabolite signaling to developing neurons in the fetal brain [33]. In an epidemiologic analysis of a large, multi-site study of pregnant women and their children, prenatal maternal microbiome was more relevant to children’s neurodevelopment than children’s microbiome in the first year of life [34]. However, early postnatal microbiomes are likely also important in early neurodevelopment. For instance, diversity and overrepresentation of several bacterial taxa within the meconium microbiome, the first stool passed after birth, has been associated with behavioral disorders at 6 months old [35]. Moreover, five-month-old infants with a family history of ASD have shown significantly lower abundance of bacteria that produce γ-aminobutyric acid (GABA), an essential neurotransmitter, before behavioral differences develop [36]. At one year of age, gut microbiome diversity, which tends to be less beneficial in early childhood than adulthood [37], was shown to be negatively associated with cognitive scores, but had no association with regional brain volumes at one and two years old [38]. These insights emphasize how sensitive brain development may be to early-life microbial influences.

Considering both Pb exposures and the gut microbiome, some evidence suggests that prenatal metal exposure may influence the developing gut microbiome, resulting in impacts on neurodevelopment. Our previous study explored the joint impact of prenatal metal exposures and childhood gut microbial signatures on depression scores in children aged 9–11 years. We found a four-component metal-microbial clique, including high Zn in the second trimester, low cobalt (Co) in the third trimester, high abundance of *Bacteroides fragilis*, and high abundance of *Faecalibacterium prausnitzii*, which was associated with significantly higher depression scores, suggesting that specific combinations of prenatal metal exposures and gut microbes may increase the likelihood of elevated depression scores in late childhood [39]. Similarly, we found that childhood gut colonization by *Akkermansia muciniphila* attenuated negative associations between combined exposure to high Zn and low Cr in the second trimester, and low Co in the third trimester of pregnancy, and depressive symptoms in childhood [40]. While these examples do not include significant findings with Pb exposure, they demonstrate the potential of the gut microbiome to interact with neuroactive prenatal metal exposures to affect neurological outcomes in late childhood, suggesting a role for microbial intervention to mitigate neurotoxicity.

**Table 1:** Summary of evidence suggesting links between early-life lead exposure, the gut microbiome, and neurodevelopment.

Citation	Population or Model	Exposure or Condition	Microbiome Findings	Neurodevelopmental or Health Implications
Liu et al., 2020 [22]	Carp model	Pb exposure in water	↓ microbial diversity; ↓ *Lactobacillus*; ↑ *Clostridium*, *Oscillibacter*	Indicates dysbiosis and inflammatory/metabolic shifts linked to Pb toxicity
Wu et al., 2016 [23]	Mouse model (early-life exposure)	Prenatal Pb exposure in drinking water	Long-term microbiome alterations persisting into adulthood	Suggests developmental windows of vulnerability
Eggers et al., 2019 [25]	Human adults	Urinary Pb concentration (low–moderate exposure)	↑ α- and β-diversity; ↑ *Proteobacteria* colonization	*Proteobacteria* linked to gut inflammation and dysbiosis [24]
Bisanz et al., 2014 [26]	Children with mixed metal exposure	Blood Pb and other metals	↑ *Gammaproteobacteria* and other taxa correlated with blood Pb	*Proteobacteria* linked to gut inflammation and dysbiosis [24]
Gao et al., 2024 [27]	Human infants	Stool Pb concentrations	Altered 114 microbial metabolic pathways	Functional disruption of microbial metabolism by Pb
Sitarik et al., 2020 [28]	Human infants	Prenatal Pb measured in teeth	Altered bacterial and fungal communities in infants (1–6 months)	Suggests prenatal Pb disrupts early microbial colonization
Eggers et al., 2023 [29]	Mother–child pairs	Maternal blood Pb during pregnancy	↓ diversity; ↓ *Ruminococcus gnavus*, *Bifidobacterium longum* at age 9–11	Indicates lasting effects of prenatal Pb on gut composition
Vuong et al., 2020 [33]	Mouse model	Maternal microbiome during pregnancy	Maternal microbiome metabolites promote fetal brain development via metabolite signaling	Demonstrates maternal microbiome’s role in fetal neurodevelopment
Sun et al., 2023 [34]	Mother–child pairs	Prenatal maternal gut microbiome	Prenatal maternal microbiome more relevant than infant microbiome in first year	Highlights prenatal microbiome importance in neurodevelopment
Naspolini et al., 2025 [35]	Human children	Early-life gut microbiome (meconium and infancy)	Associations between early microbiome composition and behavioral disorders at 6 months	Links early microbial colonization to behavioral outcomes
Zuffa et al., 2023 [36]	Human Infants with elevated ASD risk	Early-life gut microbiome	Lower abundance of GABA-producing bacteria prior to behavioral symptoms	Suggests microbial role in ASD-related neurodevelopment
Moore & Townsend, 2019 [37]	Human infants	Normal microbiome development	Infant gut microbiome diversity less beneficial than adult diversity	Provides baseline context on infant microbiome
Carlson et al., 2018 [38]	Human infants	Gut microbiome diversity and composition	Negative association of diversity with cognitive scores at 1 year	Indicates early microbial diversity may relate to cognition
Midya et al., 2024 [39]	Mother–child pairs	Prenatal metals (Zn, Co) and childhood gut microbiome	Metal–microbe clique linked to higher depression scores	Shows prenatal metals and microbes jointly affect mood
Midya et al., 2024 [40]	Mother–child pairs	Prenatal metals and childhood *A. muciniphila* colonization	*A. muciniphila* attenuates association between metals and depressive symptoms	Suggests microbiome modulation can mitigate neurotoxicity

↑ indicates an increase; ↓ indicates a decrease.

## 5. Nutritional Intervention Strategies

**Figure 2:**
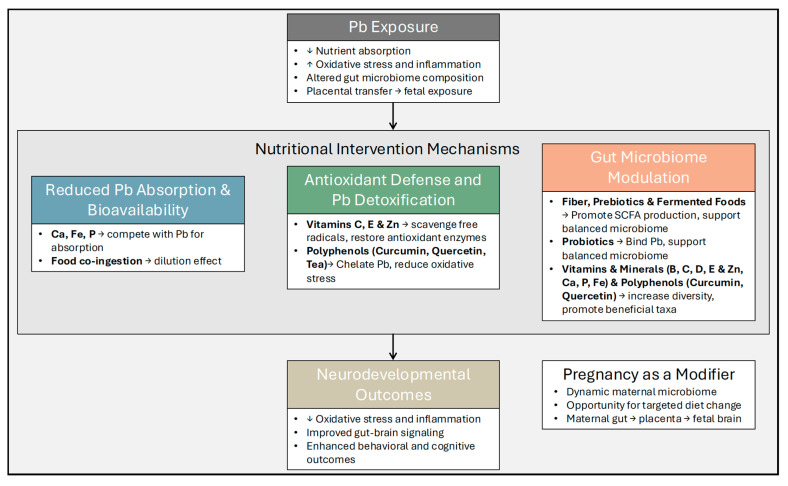
This conceptual model illustrates interconnected mechanisms through which nutritional interventions may protect against the adverse effects of prenatal lead (Pb) exposure on neurodevelopment. Dietary components act through three primary pathways: (1) reduced Pb absorption and bioavailability, where essential minerals such as calcium (Ca), iron (Fe), and phosphorus (P) compete with Pb for intestinal transporters and lower Pb uptake; (2) antioxidant defense and Pb detoxification, whereby nutrients including vitamins C and E, zinc (Zn), and plant polyphenols such as curcumin, quercetin, and tea polyphenols scavenge free radicals, chelate Pb ions, and restore antioxidant enzyme activity; and (3) gut microbiome modulation, where dietary fiber, prebiotics, probiotics, vitamins, minerals, and polyphenol-rich foods alter microbial composition, enhancing beneficial taxa such as *Bifidobacterium* and *Lactobacillus* and supporting short-chain fatty acid (SCFA) production. These pathways converge to reduce Pb-induced oxidative stress and inflammation, supporting maternal and fetal neurodevelopment. The pregnancy context (shaded background) represents a unique physiological window in which the maternal gut microbiome undergoes marked changes in composition and metabolic activity, providing an opportunity for targeted dietary interventions to enhance detoxification capacity and neuroprotective outcomes. Arrows: ↑ indicates an increase; ↓ indicates a decrease; → indicates an effect.

Targeted nutrition to reduce Pb exposure has been widely considered over the past few decades as an alternative to traditional chelation therapies, which often have substantial side effects [41]. However, few of these investigations have focused on pregnant populations. A conceptual model of proposed mechanisms is shown in Figure 2. A recent systematic review identified only seven randomized controlled trials of dietary interventions in perinatal women to reduce heavy metal toxicity, with five measuring Pb [42]. Of those, Ca supplementation was found to reduce blood Pb in both pregnant and lactating women, and jujube fruit consumption, high in several vitamins and minerals, reduced Pb in breastmilk [42].

When examining dietary interventions for Pb toxicity in other populations, total dietary consumption, as well as Ca and Fe intake, have been very well established [43]. Consuming Pb with food, particularly that supplemented with Ca or Fe, reduces Pb absorption into the bloodstream [44]. This occurs through dilution of Pb exposure with food, and competition with Pb transport by Ca and Fe. Additionally, supplementation of these essential metals can restore antioxidant enzyme function and reverse Pb-induced oxidative stress [45].

More recent investigations have shown that dietary fiber interventions and plant polyphenols like curcumin, quercetin, and tea polyphenols influence Pb toxicity by scavenging free radicals and forming complexes with Pb ions, mitigating oxidative stress, a key mechanism in Pb poisoning [46]. Probiotic supplementation of strains like *Lactobacillus rhamnosus* has also been suggested due to their promising heavy metal-binding capabilities [47]. Curcumin and quercetin treatments in rats improve behavioral performance, protect against histological alterations by Pb, and ameliorate oxidative stress [46]. Green tea administration similarly alleviates Pb-induced oxidative stress in rats [48]. Although these interventions have been primarily studied in animal models, they may hold translational potential for protecting maternal and fetal health during pregnancy, particularly by addressing Pb-induced oxidative stress, which can also influence the gut microbiome. However, it is important to note that the evidence on the safety of polyphenol consumption during pregnancy is still emerging and is highly variable depending on the specific compound, dose, timing of exposure, and health outcome investigated [49,50].

Diet is a primary driver of the gut microbiome, and some of the same dietary components found to be effective for reducing Pb toxicity have also been shown to shape the gut microbiome. For instance, curcumin administration in mice led to alterations in gut microbiota richness and diversity, along with changes in the abundance of several bacterial taxa [51]. Quercetin was given to rats that were also fed high-fat sucrose diets and was found to moderate obesity-induced changes to the gut microbiome, reducing the Firmicutes/Bacteroidetes ratio but not impacting intestinal permeability or short-chain fatty acid levels [52]. Similarly, curcumin has been shown to modulate pathogenic bacterial families in rats while also acting as an antioxidant and increasing superoxide dismutase activity [53]. In humans, a food frequency questionnaire revealed correlations between fecal microbiota and quercetin concentration, with certain bacterial families showing positive or negative associations [54]. Dietary supplementation with vitamins and minerals, such as vitamin B, C, D, and E, Zn, Ca, phosphorus (P), and Fe, has been associated with shifts in microbial populations, impacting genera like *Bifidobacterium*, and *Lactobacillus* [55]. Interventions like high-cocoa flavanol drinks and diets high in fermented foods have been shown to affect microbiota composition and diversity in human participants [56]. These findings underscore the interplay between dietary components and the gut microbiome, highlighting the potential for targeted dietary interventions to modulate the gut microbiome for improved health.

The gut microbiome during pregnancy may be a particularly important window for intervention because it undergoes significant changes in the second and third trimesters [57]. As pregnancy progresses, the maternal gut microbiome diversity decreases and shifts away from short-chain fatty acid production [57]. The wide range of physiological changes in and around the gut microbiome during pregnancy has been previously reviewed [58]. Despite these typical shifts, targeted dietary changes can still alter gut microbiome composition and function. In animal models, maternal dietary intake of Ca during pregnancy and lactation influenced offspring gut microbiome composition, with effects persisting into adulthood [59]. In observational human studies, fat, vitamins, and fiber in maternal diets are significantly associated with the maternal gut microbiome as well as neonatal gut microbiome composition and metabolic function [60]. These findings highlight the potential for diet interventions during pregnancy to beneficially shape the infant gut microbiome.

Links between prenatal nutrition and neurological health are well established, with investigations of folic acid and neural tube defects dating back to the 1960s [61]. Extensive evidence shows that supplementation of specific nutrients, like those commonly included in prenatal vitamins, has beneficial effects on neurodevelopment, while malnutrition and specific dietary patterns with high fat, highly processed foods, and low fiber have negative impacts on child neurological health and development [62]. When considering the nutritional components supportive of the gut microbiome and reduced Pb toxicity, many also improve neurological outcomes. For instance, neonatal curcumin treatment in mice alleviated autism-related symptoms, enhancing sociability, reducing repetitive behaviors, ameliorating cognitive impairments, and promoting neurogenesis [63]. Likewise, in a mouse model of ASD, prenatal quercetin administration prevented behavioral changes caused by oxidative stress in pups [64]. These consistent findings between prenatal nutritional benefits to Pb detoxification, gut microbiome, and neurological health suggest interactive mechanisms.

Some studies have specifically examined nutritional interventions to benefit the gut microbiome and neurological outcomes. For instance, the ketogenic diet significantly alters the gut microbiome to improve neurological disease symptoms and obesity, including altering the Firmicutes/Bacteroidetes ratio and reducing Proteobacteria in certain cases [65]. The gut microbiome has also been a target of dietary interventions to improve mood disorders like depression and anxiety [66]. Randomized controlled trials have demonstrated behavioral and gastrointestinal improvements with gluten-free/casein-free diets, while probiotics, containing strains like *Lactobacillus acidophilus*, *Lactobacillus rhamnosus*, and *Bifidobacteria longum*, have shown increases in beneficial gut bacteria and significant reductions in body weight, severity of autism symptoms, and gastrointestinal issues in children with ASD [67]. Although most of these nutritional interventions have been evaluated in pediatric or general populations, their potential to modulate maternal gut–brain signaling suggests relevance for promoting healthy neurodevelopment during pregnancy.

These interventions collectively underscore interconnected mechanisms aimed at improving health outcomes and mitigating neurodevelopmental disorders with influence through the gut microbiome. Vitamins, minerals, essential metals, dietary fiber, probiotic supplementation, and dietary polyphenols emerge as common interventions across these studies. Their common mechanisms are dilution and competition of toxic metals and mitigating oxidative stress, a key factor in heavy metal toxicity for both humans and gut bacteria. These dietary interventions highlight the potential of nutritional modifications and supplementation in promoting gut health, reducing oxidative stress, and potentially ameliorating symptoms of Pb-induced neurodevelopmental disorders in children. Findings from non-pregnant and animal models may not fully translate to pregnancy but nonetheless suggest potential mechanisms worth exploring in prenatal populations.

## 6. Research Gaps and Future Directions

Despite growing evidence linking prenatal Pb exposure, the gut microbiome, and offspring neurodevelopment, several gaps remain. Most existing studies are either cross-sectional or based on animal models, limiting causal inference and generalizability to human pregnancy. There is a critical need for longitudinal human studies that assess maternal microbiome composition during pregnancy and follow offspring neurodevelopmental outcomes into childhood. Additionally, few studies examined interactions between multiple metals or considered the potential modifying effects of diverse dietary patterns beyond individual nutrients. The role of nutrition, specifically, how maternal diet influences the gut microbiome during pregnancy and how these changes may interact with metal exposures to affect neurodevelopment, remains underexamined. Well-planned randomized control trials of dietary interventions, in combination with translational mechanistic studies, would have significant impact on our ability to assess causal pathways for microbiome-targeted Pb exposure mitigation.

While most mechanistic studies remain preclinical, nutritional approaches such as increasing dietary fiber, zinc, and polyphenol-rich foods may be practical for human pregnancy if guided by clinical dietary recommendations. However, further research is needed to understand how the timing, diversity, and consistency of maternal dietary exposures shape these interactions across gestation. Combining longitudinal microbiome profiling with dietary assessments, metal exposure biomarkers, and neurodevelopmental testing could yield critical insights into the biological pathways linking maternal nutrition, the gut microbiome, and Pb-induced neurotoxicity.

## 7. Conclusions

Nutritional interventions for neurodevelopment are not a novel concept, with folate supplementation being recommended since the early 1990s for people who are pregnant or trying to become pregnant [68]. However, using nutrition to target gut microbiome modification in pregnant people with high Pb exposure is not yet a common practice. Prenatal exposure to Pb poses significant risks to neurodevelopment, with emerging evidence implicating the gut microbiome in mediating these effects. Studies have revealed associations between maternal metal exposure and adverse neurodevelopmental outcomes in children, highlighting the need for interventions targeting both metal toxicity and gut microbiome dysbiosis during pregnancy. Nutritional interventions, including increased fiber consumption and supplementation of vitamins, minerals, probiotics, and dietary polyphenols, show promise in mitigating metal-induced neurodevelopmental impairments by modulating gut microbiota composition and reducing oxidative stress. Collectively, these findings underscore the interconnected mechanisms through which prenatal nutritional interventions may attenuate metal-induced adverse neurodevelopment, offering avenues for further research and development of non-pharmaceutical therapies in this critical area.

## Data Availability

No new data were created or analyzed in this study. Data Sharing is not applicable to this article.
